# Nanoparticle Transport from Mouse Vagina to Adjacent Lymph Nodes

**DOI:** 10.1371/journal.pone.0051995

**Published:** 2012-12-21

**Authors:** Byron Ballou, Susan K. Andreko, Elvira Osuna-Highley, Michael McRaven, Tina Catalone, Marcel P. Bruchez, Thomas J. Hope, Mohamed E. Labib

**Affiliations:** 1 Molecular Biosensor and Imaging Center (MBIC), Carnegie Mellon University, Pittsburgh, Pennsylvania, United States of America; 2 Institute for Complex Engineered Systems (ICES), Carnegie Mellon University, Pittsburgh, Pittsburgh, Pennsylvania, United States of America; 3 Department of Cell and Molecular Biology, Feinberg School of Medicine, Northwestern University, Chicago, Illinois, United States of America; 4 Department of Natural Science, De Sales University, Center Valley, Pittsburgh, Pennsylvania, United States of America; 5 Department of Chemistry and Department of Biological Sciences, Carnegie Mellon University, Pittsburgh, Pennsylvania, United States of America; 6 Advanced Biodevices, Princeton, New Jersey, United States of America; Université Libre de Bruxelles, Belgium

## Abstract

To test the feasibility of localized intravaginal therapy directed to neighboring lymph nodes, the transport of quantum dots across the vaginal wall was investigated. Quantum dots instilled into the mouse vagina were transported across the vaginal mucosa into draining lymph nodes, but not into distant nodes. Most of the particles were transported to the lumbar nodes; far fewer were transported to the inguinal nodes. A low level of transport was evident at 4 hr after intravaginal instillation, and transport peaked at about 36 hr after instillation. Transport was greatly enhanced by prior vaginal instillation of Nonoxynol-9. Hundreds of micrograms of nanoparticles/kg tissue (ppb) were found in the lumbar lymph nodes at 36 hr post-instillation. Our results imply that targeted transport of microbicides or immunogens from the vagina to local lymph organs is feasible. They also offer an in vivo model for assessing the toxicity of compounds intended for intravaginal use.

## Introduction

Delivering drugs or immunogens to local sites of infection offers the chance to limit disease progression while minimizing undesired systemic effects. We demonstrate here that nanoparticles can be transported to draining lymph nodes after intravaginal administration. Previous investigations have reported immunization after vaginal instillations of various immunogen-adjuvant combinations, and compared immunological responses due to intravaginal instillation with responses from other routes of administration (reviewed in Czerkinsky and Holmgren [1], [2]). Our results suggest that some of the immunity that is induced using intravaginal administration is a consequence of transport to these lymphoid organs, rather than being due to locally induced mucosal immunity. Our results also indicate that that it is feasible to target drug- or immunogen-carrying nanoparticles to local lymph nodes or to other lymphoid organs of the vagina.

## Results

### Uptake of Quantum Dots into Lymph Nodes

Mouse estrous cycles were synchronized using medroxyprogesterone, then mice were instilled intravaginally using quantum dots (Qdots,™ typically 30 pmols) having various surface coats, with or without prior treatment of the mice using 1% Nonoxynol-9 (N-9), typically 12 hr before instillation of Qdots. After instillation, mice were kept under anesthesia with hindquarters elevated for one hour. At appropriate times thereafter mice were sacrificed, lymph nodes and reproductive tracts were harvested, and Qdot transport was assessed. Most of the instilled Qdots that were transported were found in the lumbar lymph nodes. Very small quantities (one to a few Qdot aggregates) were sometimes found in the inguinal lymph nodes. All Qdots were clustered into aggregates. Transport to more distant lymph nodes was not detectable; however, the more distant lymph nodes served as a control against the possibility that some nanoparticles might have been ejected into the general circulation, possibly due to injury of the vaginal wall. **Figure S1** illustrates the parts of the vagina and the relative location of the lumbar lymph nodes. It is important to note that most of the administered dose of instilled quantum dots was probably lost due to mucus flow and to self-grooming by the mice (cp. reference [3] ).

Qdots were distributed in various ways in lymph nodes. Some nodes showed concentrations of aggregates similar to the concentrations seen at the periphery of germinal centers in animals injected intravenously [4]. In other cases, quantum dots were more concentrated at the periphery of the nodes, probably in the afferent sinuses (see **Figure S2**). In a few nodes, the quantum dots were distributed almost homogeneously.

**Figure 1:**
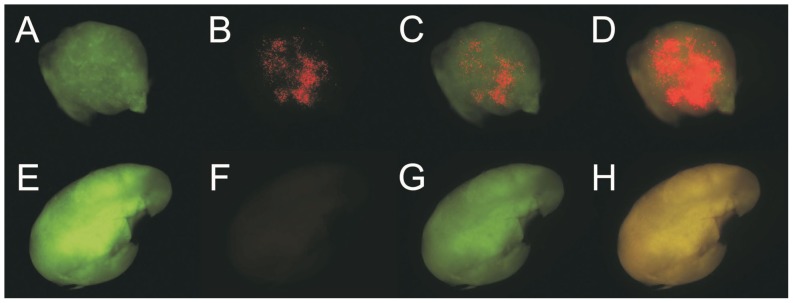
Quantum dot uptake in lumbar lymph nodes. A–D. Right lumbar lymph node, visualized 36 hr after N-9 pretreatment and 24 hr after instillation of 30 µL 1 mM polyarg-streptavidin Qdots. A. GFP spectral window (autofluorescence), false-colored green. B. Qdot spectral window, false-colored red. C. Superposition of A and B. D. Superposition of A and B with B multiplied 5x. While detection sensitivity is low at 2.5x, this lens permits a view of a whole lymph node at once. Many more quantum dots are visible than are shown in B; exposure was set to avoid saturation. E–H. Control left lumbar lymph node (mouse sham-instilled with PBS only). E. GFP spectral window (autofluorescence), false-colored green. F. Qdot spectral window, false-colored red. G. Superposition of E and F. H. Superposition of E and F with F enhanced 5x. The relatively uniform color in H indicates that the background image in F is due to autofluorescence. Imaging times were adjusted to ensure that no pixels were saturated; then all images were adjusted to correspond to uniform exposure in both red channels and the green channel. Background (assessed by averaging 4 off-image fields, one in each quadrant) was subtracted from each image.

**Figure 1** A–D shows one example of Qdot aggregates in a lumbar lymph node, imaged 24 hr after instillation of polyarginine-coupled [5], [6] 655-nm-emitting streptavidin Qdots; this mouse was pretreated using N-9 12 hr before instillation. This node shows some clustering of Qdots at the periphery of germinal centers, as normally seen in lymph nodes after intravenous injection [4], [7]. Lymph nodes from mice that had not been exposed to Qdots showed none of the fluorescent aggregates found in mice whose vaginas were instilled with Qdots. **Figure 1** E–H shows a negative lumbar node. Note that the autofluorescent areas seen in both green and red images of the negative node coincide, although it is necessary to multiply fivefold the fluorescence intensity in the 655 nm Qdot channel to demonstrate this. Thus, autofluorescence is not the source of the aggregates.

### Quantification of Uptake by Visual Inspection

Fluorescence allows visualization of Qdot aggregates at low levels. Although the background is nearly as strong as the signal, the fluorescence color of even a single Qdot aggregate is easily distinguishable at low magnification (**Figure S3**). Qdots can be identified by their spectra in whole tissues (**Figure S4**), although imaging through the whole tissue imposes a significant background autofluorescence, even when using 2-photon microscopy. We have so far failed to find any non-aggregated (blinking) Qdots when using higher magnification objectives (60x and 100x) with either confocal or 2-photon confocal imaging. In our normal surveys of lymph nodes (done using a 10x objective), we would not have seen a low level of single Qdots or very small aggregates.

Counting visible aggregates allowed facile single-blind survey of lymph nodes in our clarifying solution. This method makes no allowance for aggregate size, and the number of aggregates seen was determined by the magnification and numerical aperture of the lenses used; that is, small aggregates would be missed at low magnification. On the other hand, most aggregates were large enough to be counted easily by this method. Only one third to one half of nodes from animals instilled without N-9 pretreatment showed Qdot aggregates, typically fewer than ten aggregates per node. However, with N-9 pretreatment, most nodes showed tens to hundreds of Qdot aggregates. **Table 1** shows our method of scoring the number of quantum dot aggregates per node.

**Table 1:** Scoring nodes.

Score	Number of Qdot aggregates
0	0
1	1–10
2	11–50
3	50–100
4	>100

**Figure 2:**
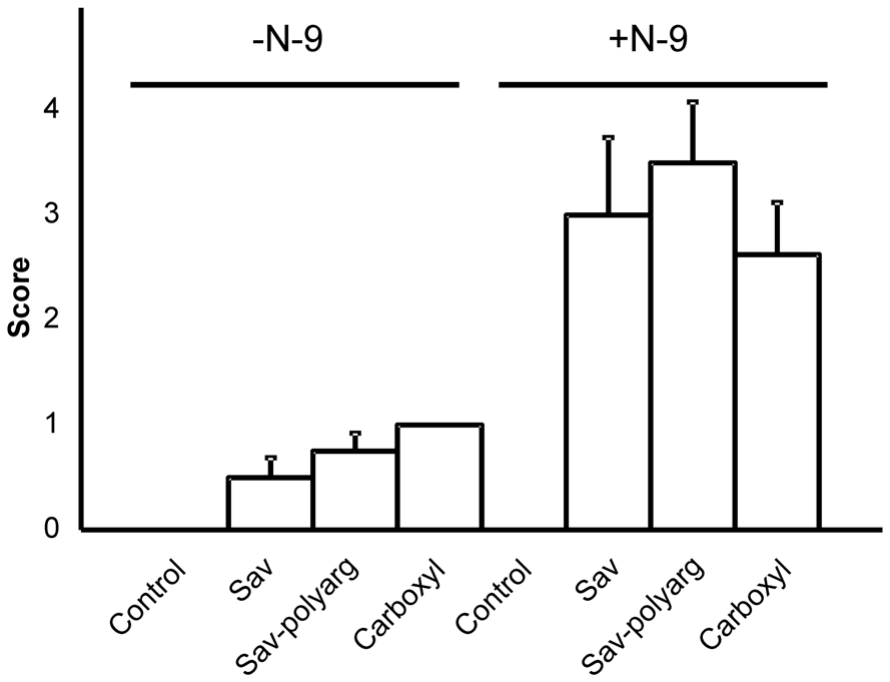
Uptake of quantum dots having three different surface coats into lumbar lymph nodes. Half of the animals were instilled using 30 µL 1% N-9 in PBS 12 h before instillation of Qdots. Either carboxyl-, streptavidin -, or polyarg-streptavidin Qdots were instilled as described in the Materials and Methods. Lumbar lymph nodes were harvested 36 hr after Qdot instillation, then evaluated by microscopy. Scoring was as described in **Table 1**. Standard errors are displayed. No standard error is given for carboxyl Qdots instilled without pretreatment, because all scored identically.

**Figure 3:**
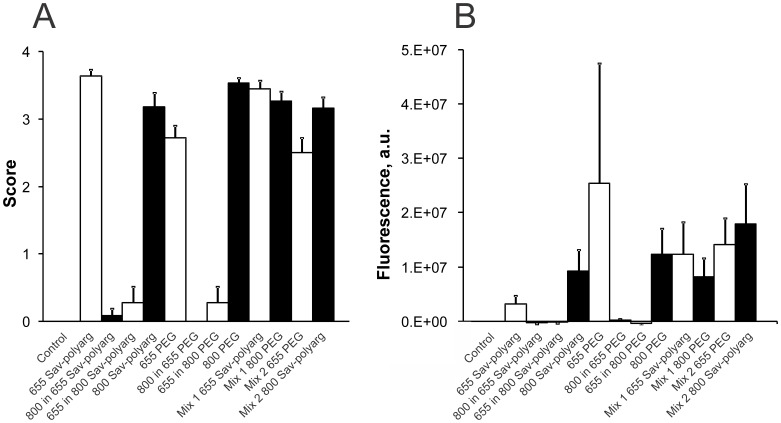
Uptake of mixed quantum dots in lymph nodes. All mice were pretreated with N-9 by instillation of 30 µL 1% N-9 in PBS 12 h before instillation of Qdots. Qdots were instilled as described in Materials and Methods, except that a total of 15 µl of quantum dots in 30 µL total volume (15 pmoles) were instilled singly, or 15 µLeach mixed to give a total volume of 30 µL(15 pmoles each). Mixtures were prepared so as to eliminate any effects due to Qdot size or emission wavelength; Mixture 1 contained 15 pmoles each 655-nm-emitting streptavidin-polyarg Qdots and 800-nm-emitting PEG Qdots, while the reciprocal Mixture 2 contained 15 pmoles each 655-nm-emitting PEG Qdots and 800-nm-emitting streptavidin-polyarg Qdots. Mice were harvested 36 hr after Qdot instillation, scored as described in **Table 1**, then integrated brightness was measured as described in Materials and Methods. **A**, Score; **B**, Integrated brightness (fluorescence was measured in arbitrary units, “a.u.”). Fluorescence due to 655-nm-emitting Qdots is shown in unfilled columns; fluorescence due to 800-nm-emitting Qdots is shown in filled columns. There are very few artifacts due to miscounts of 800-nm-emitters in nodes from mice exposed to 655 nm Qdots alone, or to miscounts of 655-nm-emitters in nodes from mice exposed to 800 nm Qdots alone (**A**); this is confirmed by measurements of integrated brightness (**B**). Standard errors are shown. Note that our scoring method collapses the differences in uptake between lymph nodes as compared to integrated brightness. This is why the standard errors shown in A appear much smaller than those in B. There are no significant differences in uptake between quantum dot having different coats or emission maxima.

We analyzed the uptake of Qdots having different surface coats. Identically administered quantum dots having carboxyl, streptavidin, or polyarg-streptavidin coats differed relatively little in uptake, both at low levels (no N-9) and at high levels (N-9 pretreatment) (**Figure 2** **)**. To confirm that there was no significant preferential uptake of Qdots having a particular surface coat, mice were instilled using mixtures of polyarg-coupled streptavidin quantum dots and PEG-coated quantum dots emitting in distinct spectral windows. As shown in **Figure 3**, lymph node uptake of Qdots having either coat was similar, whether quantum dots were instilled separately or as mixtures, and whether assessed by counting aggregates or by measuring fluorescence of whole lymph nodes (described below). We conclude that the surface coats of these quantum dots made little difference to transport.

### Quantification of Uptake by Integration Over Whole Lymph Node Images

Whole lymph nodes in clarifying medium were imaged in three spectral windows: green (GFP window) for intrinsic fluorescence, then spectral windows for 655-nm- and 800-nm-emitting quantum dots. Background and autofluorescence were estimated using data from lymph nodes from sham-instilled mice imaged in all three windows identically to the Qdot instilled mice (see Materials and Methods). After correction for background and autofluorescence, fluorescence from the whole nodes was assessed by integrating over the images. This method allows a quantitative assessment of uptake in lymph nodes that show moderate to high uptake, but fails for lymph nodes that show low numbers of quantum dot clusters (**Figure 3B**).

### Quantification of Uptake by Cadmium Analysis

Lumbar lymph nodes that showed large numbers of aggregates were subjected to z-stack integration as described in Materials and Methods, then assayed for cadmium, a major component of quantum dots, by inductively-coupled plasma-mass spectrometry (ICP-MS). Control nodes from mice that were not exposed to Qdots and samples of our clarifying solution were also assayed (the clarifying solution had no assayable cadmium content). Results from this experiment are displayed in **Table 2**. The cadmium content of normal tissues sets a limit to how low a level of uptake we can monitor using this technique.

**Table 2:** Quantification by ICP-MS and Integrated z-Stacks.

Node ID	Raw Score	Cadmium (ppm)	Integrated Brightness (a.u.)	Molarity of Cd	Molarity of QD
1	4	0.21 ppm	2.9E+9	1.9E−09	3.8E−13
2	3	0.08 ppm	6.6E+8	7.1E−10	1.4E−13
3	3	0.13 ppm	3.7E+9	1.2E−09	2.4E−13
4	4	0.18 ppm	8.1E+8	1.6E−09	3.2E−13
5	4	0.27 ppm	6.0E+9	2.4E−09	4.8E−13
6	0	<0.02 ppm	8.6E+3		
7	0	<0.02 ppm	4.1 E+3		
8	0	<0.02 ppm	3.0 E+3		
9	0	<0.02 ppm	8.7 E+3		
10	0	<0.02 ppm	7.8 E+3		

Samples 1–5 are from Qdot-instilled mice, 6–10 are from control (uninstilled) mice. All mice were pretreated using N-9. Mice were scored as described in **Table 1**. Molarity of quantum dots is computed using an average of 5,000 Cd atoms per QD. Scores were all based on single-blind counts. All numbers are rounded to two significant figures.

### Quantification of Uptake by Integration Over Z-stacks

**Figure 4:**
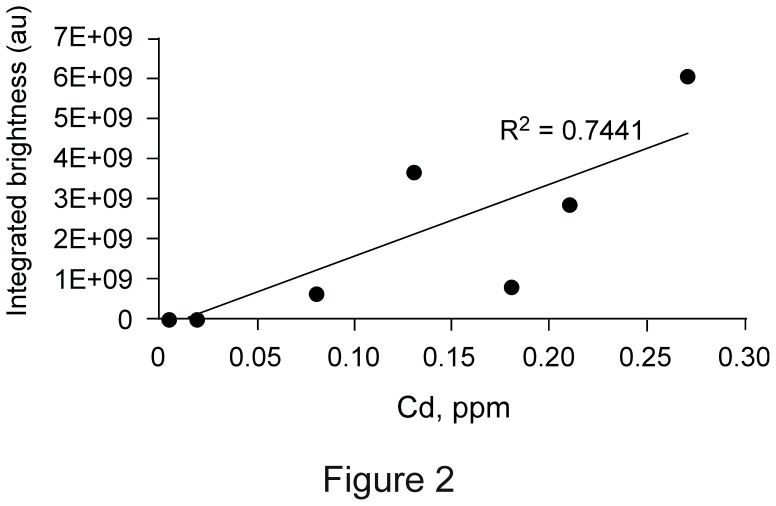
Comparison of Qdot content as assessed by integrated spot brightness with cadmium content by ICP-MS. Data were from **Table 2**.

Tiled z-stacks were prepared using a confocal microscope equipped with a 10x objective. A high-frequency filter was applied to Fourier-transformed images to select Qdot aggregates. Background was assessed using a low-frequency window (see Materials and Methods). Corrected light emission from Qdot aggregates was integrated. Ideally, this method should quantify the fluorescence emission from Qdot aggregates only (**Table 2**). However, integrated brightness showed only a weak correlation with cadmium levels (**Figure 4**). The lack of close agreement between integrated brightness and ICP-MS assayed cadmium content may be due to different levels of fluorescence quench in the aggregates or to inadequacies in the integration methods. Integrated brightness has limits similar to ICP-MS; tissue background and thickness of optical slices set a lower limit on the brightness of Qdot aggregates that can be determined.

None of the three quantification methods we employed in place of counting Qdot aggregates was adequate to assess low-level transport. In spite of its limitations, manually counting fluorescent aggregates is so far the most sensitive method for quantification over the wide range of observed lymph node uptake.

### Kinetics of Qdot Uptake

**Figure 5:**
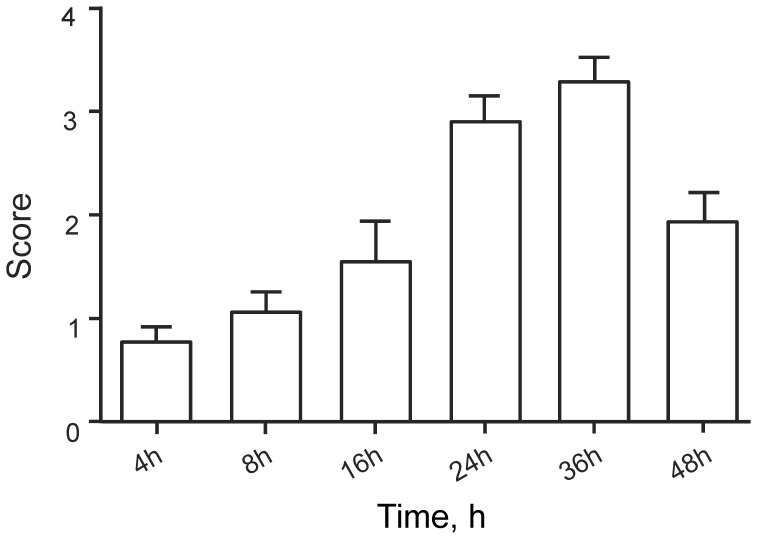
Uptake into lumbar lymph nodes at indicated times after Qdot instillation. Animals were pretreated with N-9 and instilled with polyarg-streptavidin Qdots as described in Figure 1. Five animals were analyzed for each time point and Qdot uptake was scored as in **Table 1**. SEM’s are indicated.

Animals pretreated using N-9 were instilled using polyarg-streptavidin Qdots as described above, and lymph nodes were removed at the indicated times after instillation and scored as in **Table 1**. The results are shown in **Figure 5**. Transport was very low at 4 hr post-instillation, and increased to a maximum at 24–36 hr, followed by loss at 48 hr. We do not know whether the reduction in visible aggregates at 48 hr is due to loss of the Qdots from the lymph node or to loss of fluorescence.

### Uptake of Qdots through the Vaginal Wall

The presence of Qdots in draining lymph nodes clearly demonstrated that the Qdots were penetrating the protective epithelial barriers. To gain insights into how and where the Qdots were entering the submucosa, we examined thin sections of the reproductive tract of female mice instilled using N-9 followed by Qdots. We found that Qdots readily coated the surface of the reproductive tract, typically accumulating within folds of the vagina and cervix. These are all regions protected with a squamous epithelial barrier. We did not detect Qdots in the upper reproductive tract (uterus and uterine horns). This is likely due to the protective mucus barrier, which minimizes access of materials in the vaginal lumen to the upper reproductive tract.

**Figure 6:**
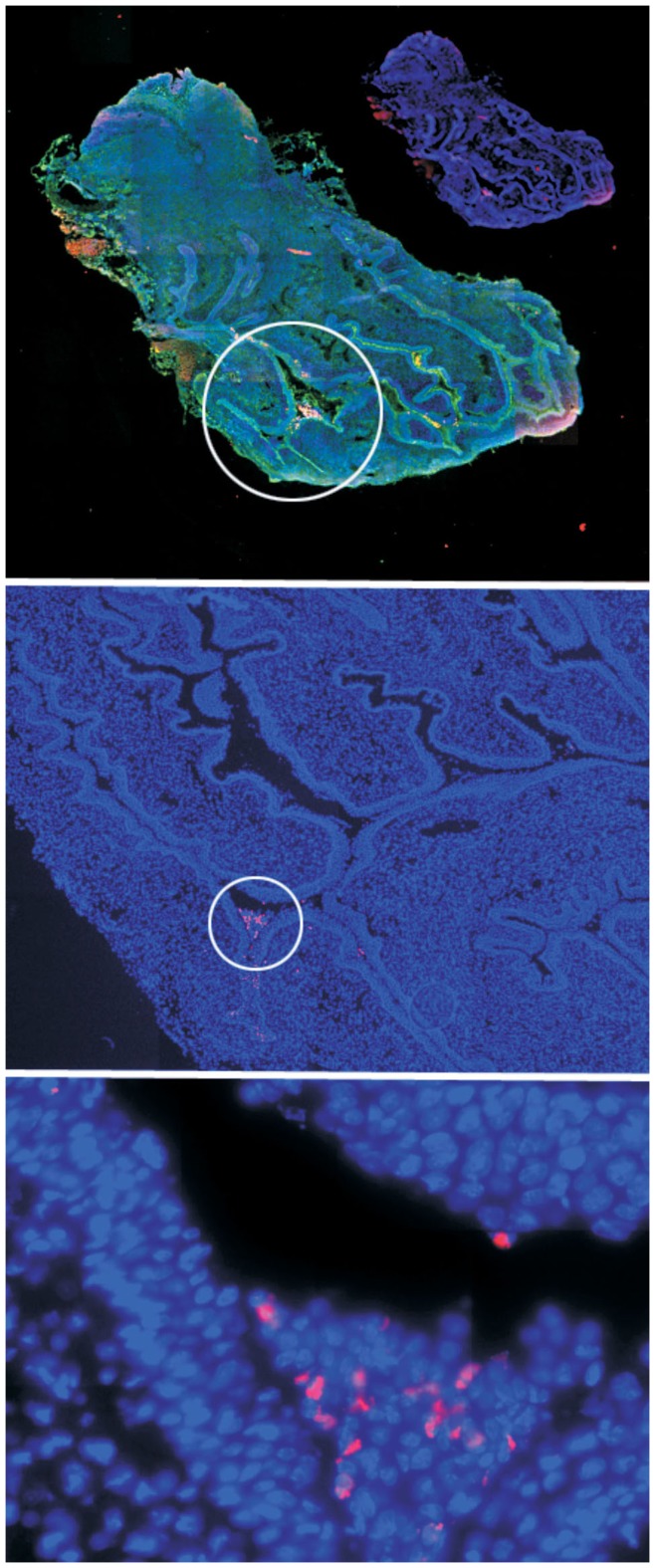
Qdots in vaginal wall close to cervix. Top to bottom, increasing magnification of circled areas. Red, Qdots; green, WGA (cell surfaces); blue, nuclei (DAPI). Note penetration of tissue in bottom figure. N-9 pretreatment and instillation were as described in Figure 1. Organs were harvested and flash-frozen without fixation 24 hr after Qdot instillation. Sectioning, staining and microscopy were as described in Materials and Methods.

**Figure 6** shows a microscope image of vaginal tissue from an N-9 pretreated animal 24 hours after Qdot exposure. The Qdots appear to be aggregated in discreet areas of folded endocervical crypts. The presence of Qdot aggregates between and possibly within epithelial cells is seen in **Figure 6**, bottom. Our initial hypothesis was that the Qdots were penetrating the epithelial barriers in small numbers over the whole surface of the mucosal barrier. However, our microscopic observations showed that the Qdots entered the submucosa by penetrating the squamous epithelial barrier at individual foci, rather than uniformly through the whole mucosa. More details and images of Qdot penetration in the submucosa are presented in **Figures S5 and S6**.

### Microscopy of Lymph Nodes

**Figure 7:**
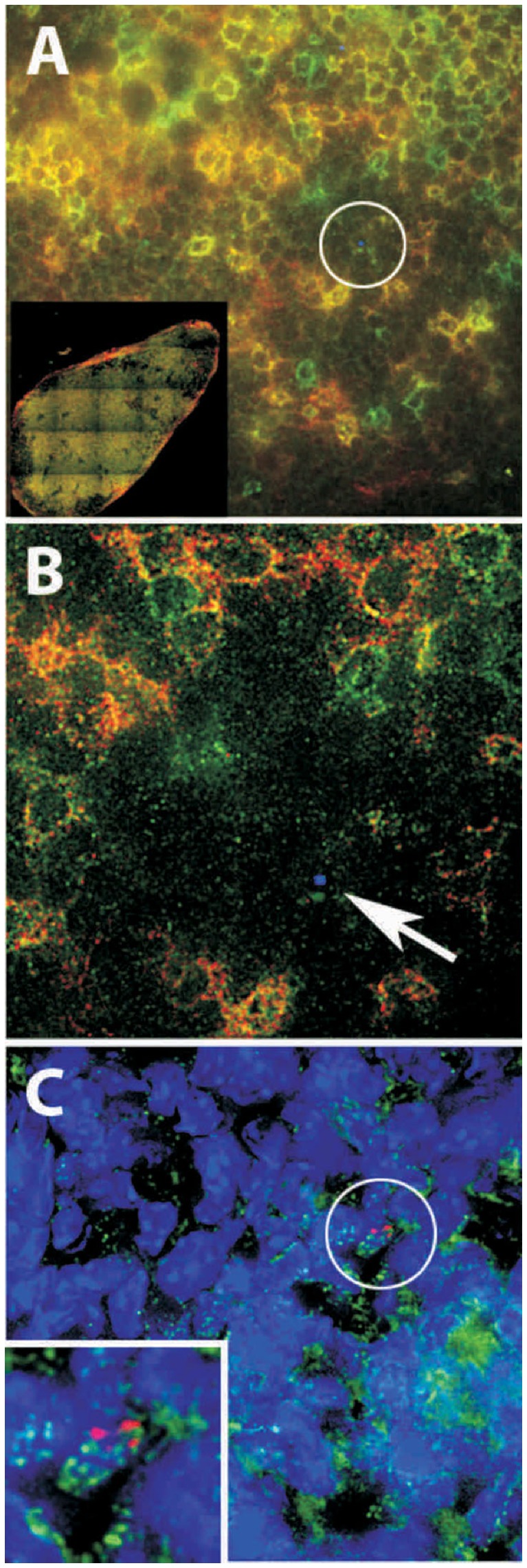
Identification of Qdots in draining lymph nodes. A. CD3 staining (green) and CD4 staining (red) in mouse lymph node. N-9 pretreatment and instillation were as described in Figure 1. Inset shows tiled panel of entire lymph node. B. Region in white circle from A, which contains a Qdot aggregate, false-colored blue, which is not associated with CD4 or CD8 cells (arrow). C. Qdots (red) associated with macrophage (CD11b, green). Blue, DAPI.

We examined draining lymph nodes from Qdot-exposed animals to determine the localization and cell association of Qdots (**Figure 7**). The Qdot clusters we found were associated with all cell types examined. Most of the Qdots appeared to be localized on or near the surface of associated cells; this argues against the idea that Qdots are picked up by a specific cell type at the mucosal barrier, and then carried to the lymph nodes. After vaginal instillation, flow cytometry of cells from disaggregated lymph nodes showed minimal association of Qdot aggregates with lymph node cells; however, using identical disaggregation methods, intravenously injected quantum dots were readily detected in macrophages and dendritic cells from lymph nodes (data not shown). Electron microscopy of quantum dot aggregates in the lymph nodes revealed a variety of sizes, with clusters of about 100 nm predominating (**Figure S7**).

## Discussion

We have demonstrated transport of Qdot nanoparticles from the vagina to local lymph nodes. The transport is directly quantifiable, without complications from amplification or reproduction as with viruses or bacteria. Several features of the transport deserve further comment.

We have no direct evidence for cell-mediated transport across the vaginal wall. In the vagina, quantum dots appear to be either free or in contact with several different cell types; we observed no association with any specific cell type. Similarly, in the lymph nodes, few of the quantum dots were within cells, or associated with any specific cell type.

Uptake of Qdots into lymph nodes was strongly enhanced by prior instillation using N-9. Previous studies showed that effects of N-9 on the mouse vagina include increased susceptibility to viral infection, exfoliation and thinning of the vaginal lining, invasion of the vaginal lumen by macrophage, and secretion of inflammatory cytokines [8]–[14] (literature reviewed in [9], [15], [16]). Apparent tissue disruption as judged microscopically was greatest at ∼2 hr post-instillation [8], but HSV infectivity was higher at 8–12 hr post treatment than at 2–4 hr [9], and major increases in inflammatory cells occurred at ∼12 hr after N-9 treatment [8]. Areas of maximum epithelial disruption do not necessarily coincide with areas of increased invasion by inflammatory cells [8]. We tested Qdot uptake at 2 hr and at 12 hr after N-9 treatment; transport was approximately fourfold greater at 12 hr than at 2 hr (data not shown). Thus, Qdot transport is greater near the time of maximum inflammation rather than the time of maximum epithelial damage.

We believe that increased permeability at the surface of the N-9 treated vagina, probably caused by the inflammatory response to N-9 [9], [17], rather than epithelial disruption, promotes Qdot penetration and transport through lymphatics. This penetration occurs at a few foci, rather than being due to a general breakdown of the vaginal wall. Our work suggests that some part of systemic immunity induced by vaginal administration of antigens may be due to transport of antigens to lymph nodes. This might explain the results of Fraillery et al. [18], who found that their virus-like particles were immunogenic after vaginal administration only if mice were pretreated using N-9.

Low-level transport occurs in the absence of N-9; we do not know whether this low-level transport takes place by a similar barrier-breakdown mechanism. For both low and high level transport, it is uncertain whether transcytosis or movement to the lymph nodes by cells that have taken up quantum dots contributes. If cellular uptake and migration are involved in transport to lymph nodes, then either the cells that took up quantum dots are very fragile, and thus destroyed by the disaggregation of lymph nodes required for FACS, or the cells expel the quantum dots. The latter possibility is consistent with our in vitro studies demonstrating exchange of quantum dots between cells; see **Figure S8**. It is also consistent with studies that show “hand-off” of HIV virus from Langerhans cells and macrophage to other cells (reviewed in Hladik and Hope [19]). However, we re-emphasize that we have no direct evidence for cellular involvement in the transport of Qdots.

Transport of bovine serum albumin, horse ferritin, and horseradish peroxidase (all FITC-labeled) [20], fluorescent-labeled exogenous lymphocytes [21] and fluorescent-labeled normal and virus-infected host cells [22] from the vagina to the iliac lymph node has been reported. We rarely find transport of quantum dots to this node. Of course, transport of proteins and cells need not be similar to transport of quantum dots.

Nanoparticles offer potential improvements in selective drug delivery in the vagina (reviews: [23], [24]). Transport of nanoparticles through vaginal mucosa to the vaginal epithelium is an active area of research. PLGA (poly lactic co-glycolic acid) nanoparticles containing siRNA [25] and RANTES [26] have been administered intravaginally and shown to be more effective in vivo than the corresponding therapeutics administered directly [23]). However, vaginal mucus limits the diffusion and migration of macromolecules [27], [28] and nanoparticles [29]–[34]. Large nanoparticles (>100 nm) were significantly retarded unless coated so as to minimize interaction with mucus (e.g., by coating with PEG). Retention in the vagina was explored quantitatively by Cu et al. [3], who found that PEG- and avidin-coated PLGA nanoparticles were retained for longer periods in the vagina than uncoated PLGA nanoparticles, with PEG-coated nanoparticles showing the most retention. All three particle types penetrated the vaginal wall and were found in association with mucosal and submucosal cells. In a subsequent report, Ensign et al. [26] found that mucus-penetrating PEG-coated nanoparticles were retained more uniformly and in higher amounts on the surface of and within vaginal epithelium than uncoated nanoparticles, and that PEG-coated nanoparticles containing acyclovir monophosphate were more effective against Herpes simplex virus than the soluble drug. Given these previous observations, the results of our experiments using PEG-coated and polyarginine coated streptavidin Qdots, which showed essentially equal uptake into lymph nodes, are therefore unexpected. However, (1) the nanoparticles we used are different in size and composition from those used by others; and (2) we are measuring uptake into lymph nodes, not diffusion through mucus, binding to the vaginal epithelium, or retention in the vagina. The lack of any strong effect of quantum dot coats on transport to lymph nodes recalls our earlier finding of minimal effects of quantum dot coats on sentinel lymph node uptake [7].

Quantum dots have been used to follow lymph node uptake and to map lymph node drainage basins in several animal models [7], [35]–[43]. Our work shows again the usefulness of these nanoparticles as sensitive probes for assessing transport in vivo.

In our experiments, almost all quantum dots in lymph nodes were seen in large aggregates. Quantum dots aggregate after polyarginine coupling [5], [6]; we would expect polycationic quantum dot aggregates to bind to negatively charged mucus proteins and aggregate still further. That both streptavidin and carboxyl Qdots would aggregate in presence of mucus was also not surprising, given that carboxyl surfaced nanoparticles [31] and avidin surfaced nanoparticles [3] interact with vaginal mucus. What was unexpected is the finding of similar aggregates of PEG-coated Qdots in the lymph nodes. That transport of large aggregates is possible presages well for potential drug- or immunogen-carrying nanoparticles.

Even though there are many in vitro assay systems for cervicovaginal toxicity [44], there is a critical need for in vivo assays [45]. Demonstration of the very strong enhancement of nanoparticle transport by N-9 suggests that the disruptive effect of other agents might be similarly tested. Thus, monitoring nanoparticle migration from the urogenital tract to lymph nodes may allow an in vivo screen for compounds intended for intravaginal use.

To our knowledge, this is the first report of nanoparticle transport across the vaginal mucosa to lymph nodes. Whether enhanced delivery of nanoparticles from the vagina to local lymph nodes is feasible without the use of N-9 remains to be determined. However, our results show that at least under some conditions, sufficiently large quantities of material can be delivered to lymph nodes to allow a new drug delivery strategy for prophylactic and therapeutic applications.

## Materials and Methods

### Reagents

Except as noted, all reagents were purchased from Sigma-Aldrich and used without further purification. Quantum dots were purchased from Invitrogen (www.invitrogen.com); Hypaque (50% solution of sodium diatrizoate; outdated Hypaque intravenous solution) was originally obtained from Winthrop Laboratories, and was a kind gift from the VA Hospital, Pittsburgh PA; medroxyprogesterone acetate was from Greenstone LLC, Prepack, NJ; biotinylated poly-D-arginine (nonamer) was synthesized by New England Peptide, 65 Zub Lane Gardner, MA; paraformaldehyde (16% in water) was purchased from Electron Microscopy Sciences, Hatfield, PA. Optical filters were from Chroma Technology, Bellows Falls, VT.

### Animals

All procedures were approved by the Carnegie Mellon University IACUC (approval number AS12-034). For instillations, mice were anesthetized using either isofluorane, 1.5% in 2∶1 oxygen-nitrous oxide, or sodium pentobarbital, 80 mg/kg, i.p. (doses were adjusted to effect, if necessary.) Mice were sacrificed by anesthesia using isofluorane, followed by an overdose of pentobarbital i.p.

1. *Estrous cycles* were synchronized by administration of medroxyprogesterone acetate (Greenstone LLC, Peapack, NJ, 07977). The solution is provided at 150 mg/ml; this was diluted threefold in PBS, and 3 mg/animal (60 µL) were given sc in the flank at day −7 and day −3 before vaginal instillation. This protocol ensured that the mice would be more uniform than if estrous cycles were random and that new epithelium was exposed [8], [9].
2. Where appropriate, *mice were pretreated using Nonoxynol-9* (N-9, 1% w/v in PBS, 30 µL), instilled intravaginally 12 hr before quantum dots were instilled [9]. Instillation was performed without penetrating the vagina by pipetting the dose gently down one side of the opening, eliminating any possibility of physical damage. After mice were instilled, anesthesia was maintained, and their hindquarters were kept elevated for 1 hr, after which the mice were returned to their cages (cp. reference 5.)
3. *Quantum dots were instilled* in the same fashion. Dose was typically 30 µL, 1 µM quantum dots, for a total of 30 pmoles of quantum dots.

### Quantum Dots

Before instillation, streptavidin quantum dots (Invitrogen, Life Technologies, Grand Island, NY) were coupled to a 50x molar excess of biotinylated polyarginine nonamer [5], [6]. Coupling was for 10 min at room temperature, then 1/9 volume of 1.5 M NaCl was added to yield an approximately isotonic solution. These polyarg-quantum dots tend to aggregate in solution; they are internalized by cells in vitro as large aggregates [5], [6], [46]. The large aggregates can be seen in a low-power microscope, making survey of tissues easier. Unconjugated streptavidin-quantum dots were similarly made up and instilled; these also appeared as aggregates when observed in lymph nodes. Carboxy- and PEG-quantum dots were adjusted to the desired concentration by dilution in 0.01 M sodium borate, then 1/9 volume of 1.5 M NaCl was added. Again, the quantum dots were seen as aggregates.

### Tissue Preparation

#### 1. Whole lymph nodes

Lymph nodes were clarified, so that localization of small amounts of quantum dots could be observed and quantified. Two methods were used:

- Murray’s clearing solution. In brief, after animal organs were harvested, the organs were fixed and dehydrated through a graded alcohol sequence, extracted with hexane, then infiltrated with a mixture of one part benzyl alcohol and two parts benzoyl benzoate [47], [48]. This method gave excellent transparency, but was tedious, required several days, and quantum dots proved unstable in the medium, losing their fluorescence over a few days.
- We therefore devised another method, in which lightly fixed lymph nodes (2% paraformaldehyde in PBS, 4 hr at r.t, followed by overnight wash with 20% ethanol) were infiltrated using a medium consisting of 1∶1::glycerol:50% w/v Hypaque-0.05 M Tris-Cl-0.001 M ZnCl_2_-0.0005 M tributyl phosphine, final pH 7.5. This solution produced adequate transparency, required only a day for infiltration, and quantum dots were more stable (weeks). For convenience in preparation, we used outdated 50% Hypaque intravenous solution from Winthrop laboratories in place of freshly dissolved Hypaque; buffer and supplements described above were added in a minimal additional volume (<1% of the i.v. solution volume), and the solution was then made 1∶1 with glycerol. Preservation of tissue morphology is not as good as obtained using Murray’s solution, but was adequate for our purposes (cp. Reference [49]).

#### 2. Sections

Tissues were frozen on dry ice in TBS Tissue Freezing Medium (Triangle Biomedical Sciences, Durham, NC), then sectioned 12 µm thick and stained for Anti Iba1 (Wako Chemicals USA, Inc., Richmond, VA), CD4 (Abcam, Cambridge, MA), or CD3 (Abcam). Secondary antibodies, labeled using Rhodamine Red X (Jackson ImmunoResearch, West Grove, PA) and FITC (Jackson ImmunoResearch), were utilized. Hoechst DAPI (Invitrogen) was used for staining DNA, and wheat germ agglutinin (WGA, Invitrogen) for highlighting cellular glycoproteins.

### Microscopy

#### 1. Aggregate identification

Lymph nodes were visualized using an Axiomat 2 microscope equipped with an Apotome unit (Carl Zeiss, Jena, Germany). Lymph nodes were surveyed at 2.5x (which allowed whole lymph nodes to be surveyed in one field) 5x, and 10x using fluorescence excitation at 450 nm (450x50 excitation filter, Chroma) with emission windows at 655 nm (654x24 emission filter, Chroma) and 800 nm (Chroma Qdot 800 filter set, product number 32021). Note: the currently available Qdot 655 40 nm emission filter set from Chroma, product number 32012, also works well. Autofluorescence was estimated from emission in the green (Chroma GFP filter set 49002). Lymph nodes were assessed for quantum dots in random order; the observer was not told which lymph nodes were presented (single-blind). Quantification was performed by counting the number of observed quantum dot aggregates in a node.

#### 2. Fluorescence assessment using integration over entire lymph nodes

Images of whole lymph nodes were prepared using identical exposure conditions throughout an experimental series, then a region of interest (ROI) encompassing the entire lymph node, but as little as possible of the rest of the image, was prepared for each lymph node. Background was subtracted from images in each of the three image windows described above. After background subtraction, control lymph nodes from uninstilled animals were used to establish an average ratio of autofluorescence in the red (655 nm, 800 nm) channels to autofluorescence in the green (GFP, 520 nm) channel. This factor was used for green-channel based subtraction of autofluorescence from the 655 nm and 800 nm spectral windows (cp Figure 1, E–H, which illustrate autofluorescence in red and green channels). Finally, remaining fluorescence in the 655 nm and 800 nm spectral windows was summed over the ROI for each image.

#### 3. Confocal microscopy

Performed using a Zeiss LSM 510 Meta/UV DuoScan Inverted Spectral Confocal Microscope (Carl Zeiss, Jena, Germany.) Tiled z-stacks were acquired using 5x and 10x objectives. The confocal images were then used to perform computational analysis of the amount of fluorescence. Since lymph nodes exhibit considerable autofluorescence, the first task was to eliminate this from our calculations. The Fourier transform of each image was taken and the derivative in frequency space was calculated. This eliminated low frequency signals and upon calculating the inverse Fourier transform, the image no longer had the “haze” of the autofluorescence from the lymph node itself [50]–[54]. After the autofluorescence removal, it is easier to identify aggregates of Q-dots. Qdot aggregates were then selected using a high-frequency filter. The net fluorescence for each image was assessed by summing the fluorescence intensity in the aggregates, then compared to the results from ICP-MS assay.

#### 4. Two-photon microscopy

Performed using an Axio Observer Z1 microscope with an LSM 510 Meta NLO confocal scan head (Carl Zeiss, Jena, Germany) coupled to a Chameleon Ultra-II femtosecond titanium sapphire laser (Coherent Laser Inc., Santa Clara, CA) as the excitation source. The Zeiss META detector was used for spectral analysis.

#### 5. Frozen sections

Images were obtained by deconvolution microscopy on a DeltaVision RT system (Applied Precision, Issaquah WA) collected on a digital camera (CoolSNAP HQ; Photometrics, Tucson, AZ) using 100x, 40x, and 10x oil objectives. Z-scan stacks were collected over 15 µm for each image field and image analysis was performed with the softWoRx software (Applied Precision).

### Cadmium Analysis

For quantification, cadmium in highly-labeled lymph nodes was assayed using inductively-coupled plasma-mass spectrometry (ICP-MS); assays were performed blind by EAI (Elemental Analysis Incorporated, Lexington, KY.) The company knew neither the purpose of the experiment nor the order of lymph nodes given. Because of the significant level of cadmium in normal lymph nodes, it is not possible to determine quantum dot content in low-fluorescence nodes using ICP-MS.

## Supporting Information

Figure S1 **Mouse female reproductive tract**, outline drawn from photo of necropsied mouse. Significant parts are labeled. Position of the lumbar lymph nodes is indicated. The cervix, internal, is indicated by light lines. Note that the interior of the vagina is actually highly convoluted (not shown here.) The lumbar lymph nodes are anterior and slightly dorsal to the female reproductive tract.
(TIF)

Figure S2 **Distribution of Qdots in lymph nodes. Three perspectives.** The smooth curve is the approximate outline of the node. Arrows indicate rotations of 30° (middle image) and 80° (right image) in the sense indicated by the arrows. From a mouse pretreated using N-9, then instilled using polyarg-streptavidin Qdots as described above, fixed and infiltrated as described in **Materials and Methods**. Lymph node removed 24 h post instillation. Z-stacks were made using the Apotome-equipped Zeiss Axiomat 2 microscope. The figure shows Qdot clusters in a lumbar lymph node from a z-stack taken at 10x. Note the wide distribution of cluster sizes and the location of a plurality of the Qdots along edges of the node. This is not consistent from node to node.
(TIF)

Figure S3 **Single cluster detected in lumbar node** from mouse instilled using 655 nm quantum dots. Image plane about halfway through the node. 5x objective, false-colored red. Manual scans of lymph nodes by the eye are more rapid than and as sensitive as automated and computerized scans. **Figure S3** shows one example of detecting a single cluster of quantum dots in a lumbar lymph node. As demonstrated using the 2-photon spectral microscope (see **Figure S4**), so with the eye; there is no difficulty in distinguishing 655 nm quantum dot clusters from background or occasional bright background spots.
(TIF)

Figure S4 **Identity of Qdots confirmed spectrally.** The figure shows an image of Qdot clusters taken using a two-photon excitation source (left). In this experiment, 525 nm-emitting Qdots were administered by injection, while 655 nm-Qdots were instilled intravaginally, then the lumbar lymph nodes were harvested 24 h later. Images and spectra for both colors of Qdot clusters are indicated. Left, microscope image from a single plane in a lumbar lymph node, showing spectrally unmixed 525 nm (green and light green) and 655 nm (red and purple) Qdots. Right, spectra from the indicated Qdots indicated by the same colors as the corresponding circles in the microscope image. Note the 655 nm emission wavelength and narrow emission spectrum of the red Qdots (purple and red circles (left) and similarly colored spectral lines (right)). Strong green background fluorescence alters the normally tight spectra from the green Qdots (light green and green circles (left) and spectral lines (right.)) See **Materials and Methods** for details of 2-photon microscopy.
(TIF)

Figure S5 **Qdot penetration of vaginal squamous epithelial barriers.** A. Location of Qdots in a mouse vagina 24 hours after instillation. B. Multiple foci of penetration of Qdots through the squamous epithlelium. Staining as in Fig. 5 in the main text. As in **Figure 5** of the main text, Qdots are penetrating through discreet foci in the squamous epithelium. We have examined the female reproductive tract of 6 mice. Regions of Qdot penetration were found in all mice, but the number of foci varied considerably from animal to animal. In half of the animals analyzed to date, the regions of Qdot penetration of the epithelia were relatively easy to find. Foci were observed in almost every section of tissue examined. In the other cases, there were fewer foci of Qdot penetration and many sections had to be examined to identify foci. This heterogeneity of epithelial penetration by Qdots is consistent with the between-animal variability of the number of Qdots observed to reach local lymph nodes.
(TIF)

Figure S6 **Qdots in the submucosa of mouse cervix 24 hours after instillation.** Green, CD4; red, Qdots; blue, DAPI. To gain further insight into the mechanism of penetration of the Qdots into the epithelium, we examined the submucosal regions where they were located. An example of such localization of Qdots in the submucosa is shown. Typically, aggregates of Qdots were observed; although we had anticipated that Qdots would always be found associated with a cell type, such as macrophages, Langerhans cells, or dendritic cells, such events were only rarely seen in the mucosal epithelium. In this figure, some aggregates appear to be associated with CD4-positive cells, others are not. This observation contrasts with our preconceptions, and suggests that some small regions (foci) of the epithelium do not provide effective barrier function; thus, materials from the lumen may reach the submucosa without specific cellular transport.
(TIF)

Figure S7 **Electron micrograph of Qdot cluster imaged in midplane.** Thin section taken from a lymph node. The lymph node was fixed and embedded in Epon without staining, then 20 nm sections were cut and imaged using an electron microscope. This aggregate is typical of those seen so far. Diameter of the cluster is approximately 120 nm.
(TIF)

Figure S8 **Transfer of Qdots between dendritic cells (DC’s).** To test the possibility that Qdots can be passed among cells as they are transported through the tissue to eventually reach the draining lymph nodes, rather than just drifting between cells, we used monocyte-derived dendritic cells (MDDCs) as a model for the passing of Qdots between cells. One aliquot of activated MDDC was stained with carboxyfluorescein diacetate, succinimidyl ester (CFSE, Invitrogen, Molecular Probes, Eugene, OR), washed, and then mixed with Qdots for 15 minutes, followed by washing with PBS. The Qdot-loaded DCs were then mixed with unlabelled DCs for 15 minutes before plating onto coverslips. The results in Figure S8 show that the Qdots were transferred from one labeled population to the other during co-culture. Note that in Panel B, CFSE emission is not shown in order to reveal the remaining Qdots in the CFSE-stained cell. Thus, one way that the Qdots move through the submucosa may be passage between cells within the tissue. Green, CFSE-labeled DC’s; red, Qdots; Blue, DAPI. Transfer of the Qdots to the unlabeled DC’s is readily apparent. Panel A shows three colors while panel B (no CFSE displayed) shows that the CFSE labeled cell DC shown in A still retains abundant Qdots.
(TIF)
