# Supplementary figures and images for: Nanoparticle Transport from Mouse Vagina to Adjacent Lymph Nodes
Source: PLoS One. 2012 Dec 21;7(12):e51995. doi: 10.1371/journal.pone.0051995 (PMC3528720; doi:10.1371/journal.pone.0051995)

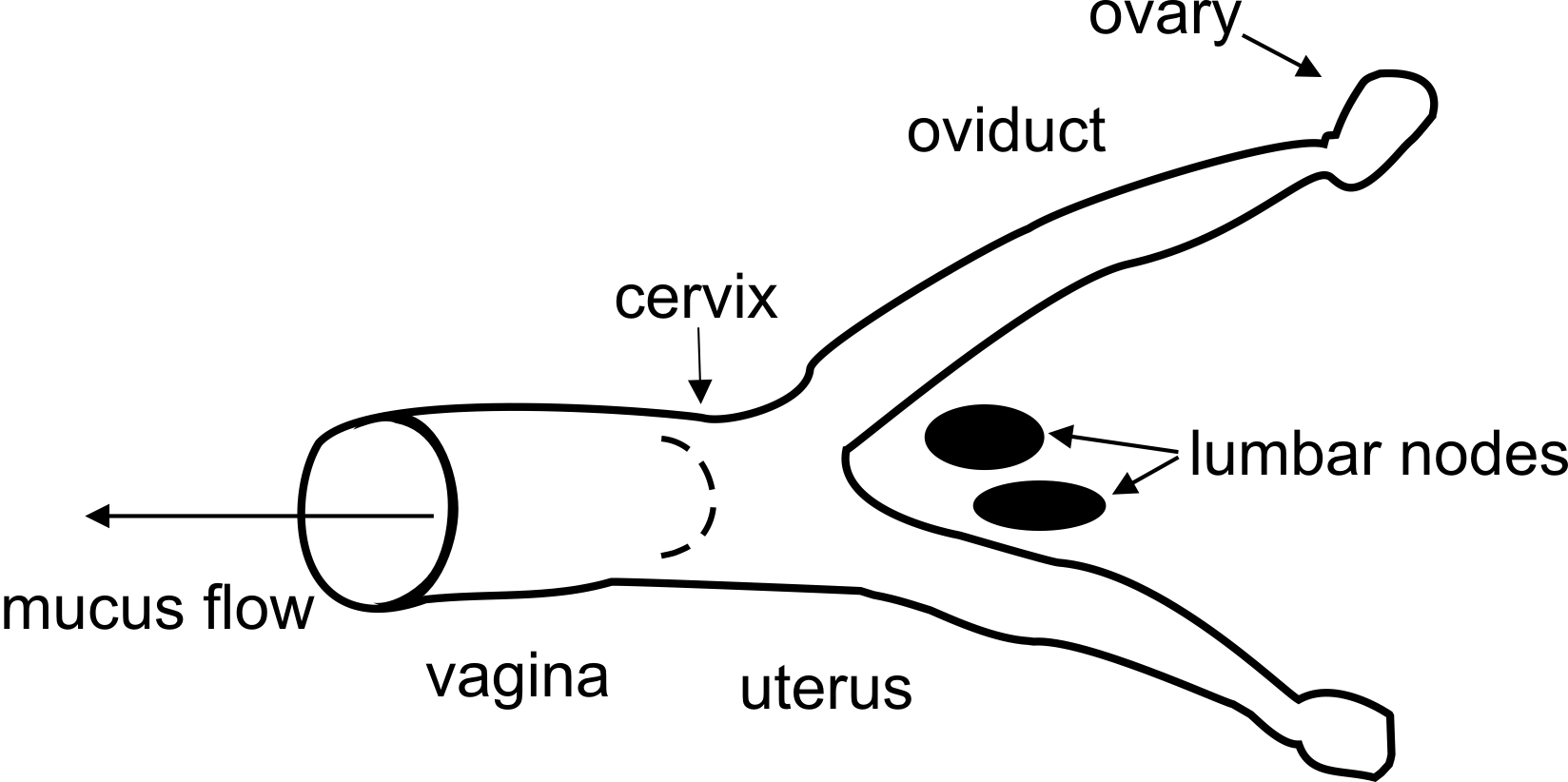

Supplement: Figure S1 — Mouse female reproductive tract, outline drawn from photo of necropsied mouse. Significant parts are labeled. Position of the lumbar lymph nodes is indicated. The cervix, internal, is indicated by light lines. Note that the interior of the vagina is actually highly convoluted (not shown here.) The lumbar lymph nodes are anterior and slightly dorsal to the female reproductive tract. (TIF) [file pone.0051995.s001.tif]

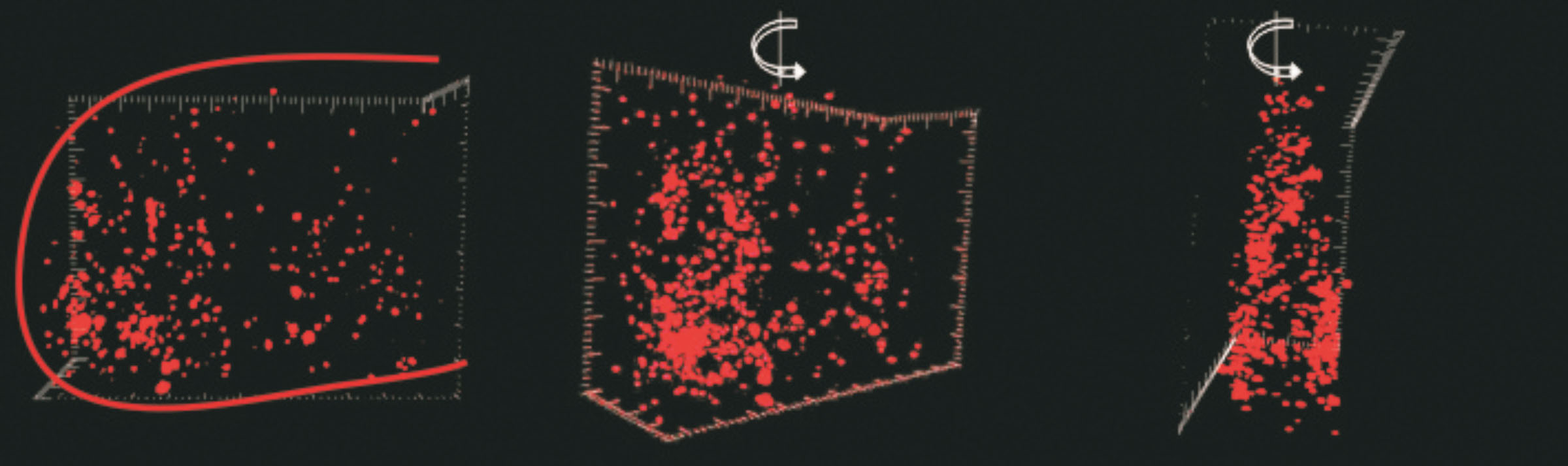

Supplement: Figure S2 — Distribution of Qdots in lymph nodes. Three perspectives. The smooth curve is the approximate outline of the node. Arrows indicate rotations of 30° (middle image) and 80° (right image) in the sense indicated by the arrows. From a mouse pretreated using N-9, then instilled using polyarg-streptavidin Qdots as described above, fixed and infiltrated as described in Materials and Methods. Lymph node removed 24 h post instillation. Z-stacks were made using the Apotome-equipped Zeiss Axiomat 2 microscope. The figure shows Qdot clusters in a lumbar lymph node from a z-stack taken at 10x. Note the wide distribution of cluster sizes and the location of a plurality of the Qdots along edges of the node. This is not consistent from node to node. (TIF) [file pone.0051995.s002.tif]

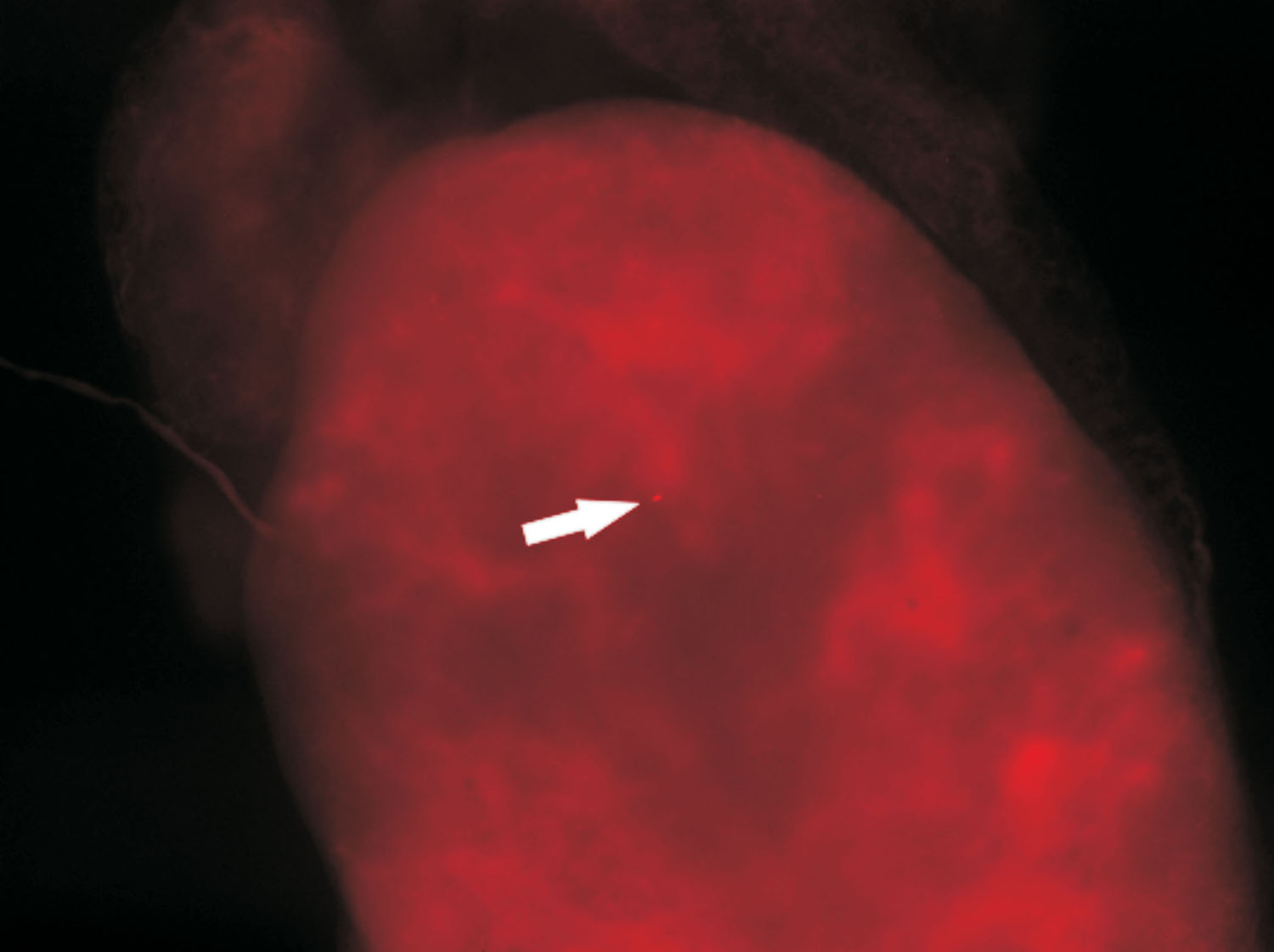

Supplement: Figure S3 — Single cluster detected in lumbar node from mouse instilled using 655 nm quantum dots. Image plane about halfway through the node. 5x objective, false-colored red. Manual scans of lymph nodes by the eye are more rapid than and as sensitive as automated and computerized scans. Figure S3 shows one example of detecting a single cluster of quantum dots in a lumbar lymph node. As demonstrated using the 2-photon spectral microscope (see Figure S4), so with the eye; there is no difficulty in distinguishing 655 nm quantum dot clusters from background or occasional bright background spots. (TIF) [file pone.0051995.s003.tif]

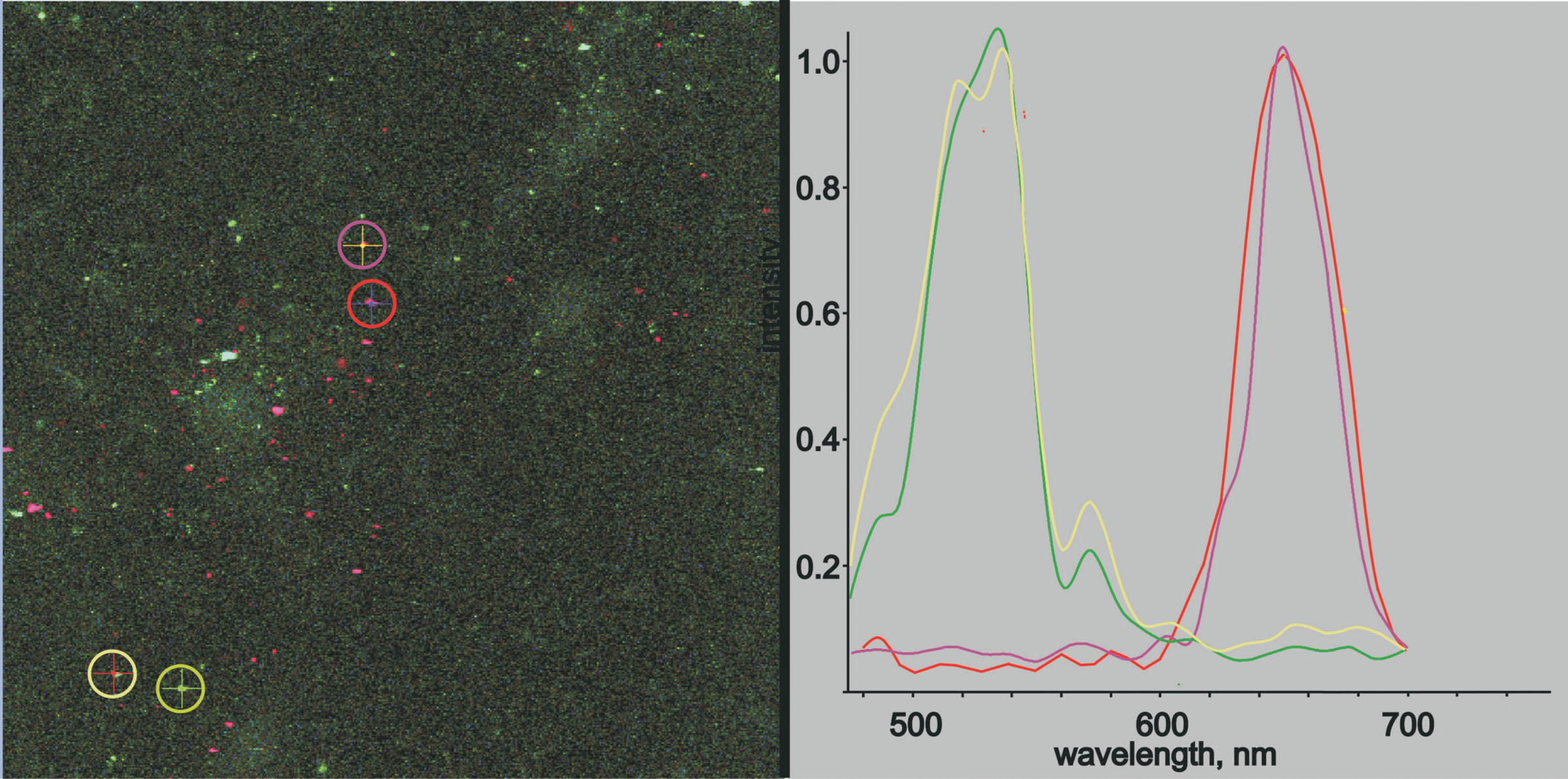

Supplement: Figure S4 — Identity of Qdots confirmed spectrally. The figure shows an image of Qdot clusters taken using a two-photon excitation source (left). In this experiment, 525 nm-emitting Qdots were administered by injection, while 655 nm-Qdots were instilled intravaginally, then the lumbar lymph nodes were harvested 24 h later. Images and spectra for both colors of Qdot clusters are indicated. Left, microscope image from a single plane in a lumbar lymph node, showing spectrally unmixed 525 nm (green and light green) and 655 nm (red and purple) Qdots. Right, spectra from the indicated Qdots indicated by the same colors as the corresponding circles in the microscope image. Note the 655 nm emission wavelength and narrow emission spectrum of the red Qdots (purple and red circles (left) and similarly colored spectral lines (right)). Strong green background fluorescence alters the normally tight spectra from the green Qdots (light green and green circles (left) and spectral lines (right.)) See Materials and Methods for details of 2-photon microscopy. (TIF) [file pone.0051995.s004.tif]

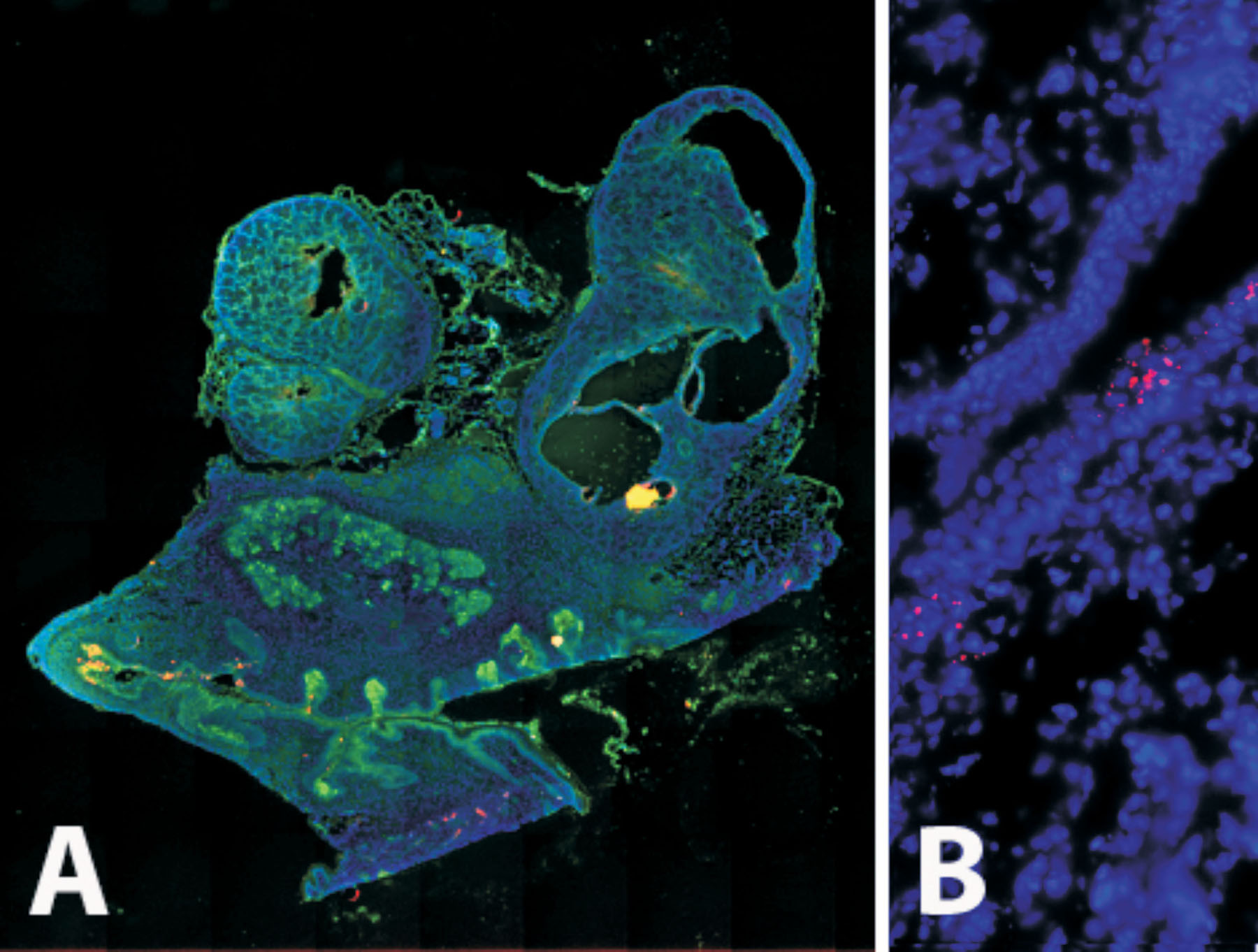

Supplement: Figure S5 — Qdot penetration of vaginal squamous epithelial barriers. A. Location of Qdots in a mouse vagina 24 hours after instillation. B. Multiple foci of penetration of Qdots through the squamous epithlelium. Staining as in Fig. 5 in the main text. As in Figure 5 of the main text, Qdots are penetrating through discreet foci in the squamous epithelium. We have examined the female reproductive tract of 6 mice. Regions of Qdot penetration were found in all mice, but the number of foci varied considerably from animal to animal. In half of the animals analyzed to date, the regions of Qdot penetration of the epithelia were relatively easy to find. Foci were observed in almost every section of tissue examined. In the other cases, there were fewer foci of Qdot penetration and many sections had to be examined to identify foci. This heterogeneity of epithelial penetration by Qdots is consistent with the between-animal variability of the number of Qdots observed to reach local lymph nodes. (TIF) [file pone.0051995.s005.tif]

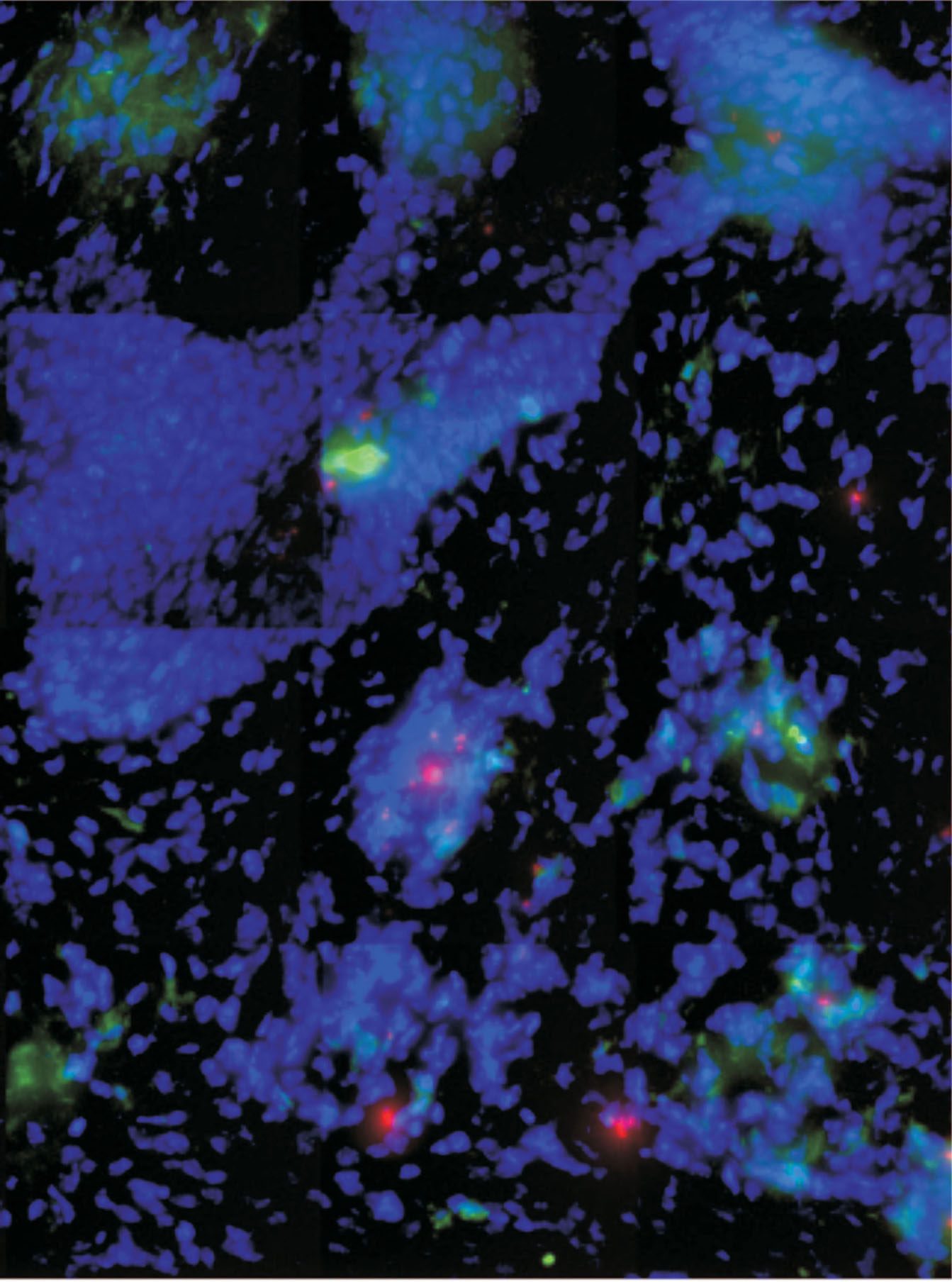

Supplement: Figure S6 — Qdots in the submucosa of mouse cervix 24 hours after instillation. Green, CD4; red, Qdots; blue, DAPI. To gain further insight into the mechanism of penetration of the Qdots into the epithelium, we examined the submucosal regions where they were located. An example of such localization of Qdots in the submucosa is shown. Typically, aggregates of Qdots were observed; although we had anticipated that Qdots would always be found associated with a cell type, such as macrophages, Langerhans cells, or dendritic cells, such events were only rarely seen in the mucosal epithelium. In this figure, some aggregates appear to be associated with CD4-positive cells, others are not. This observation contrasts with our preconceptions, and suggests that some small regions (foci) of the epithelium do not provide effective barrier function; thus, materials from the lumen may reach the submucosa without specific cellular transport. (TIF) [file pone.0051995.s006.tif]

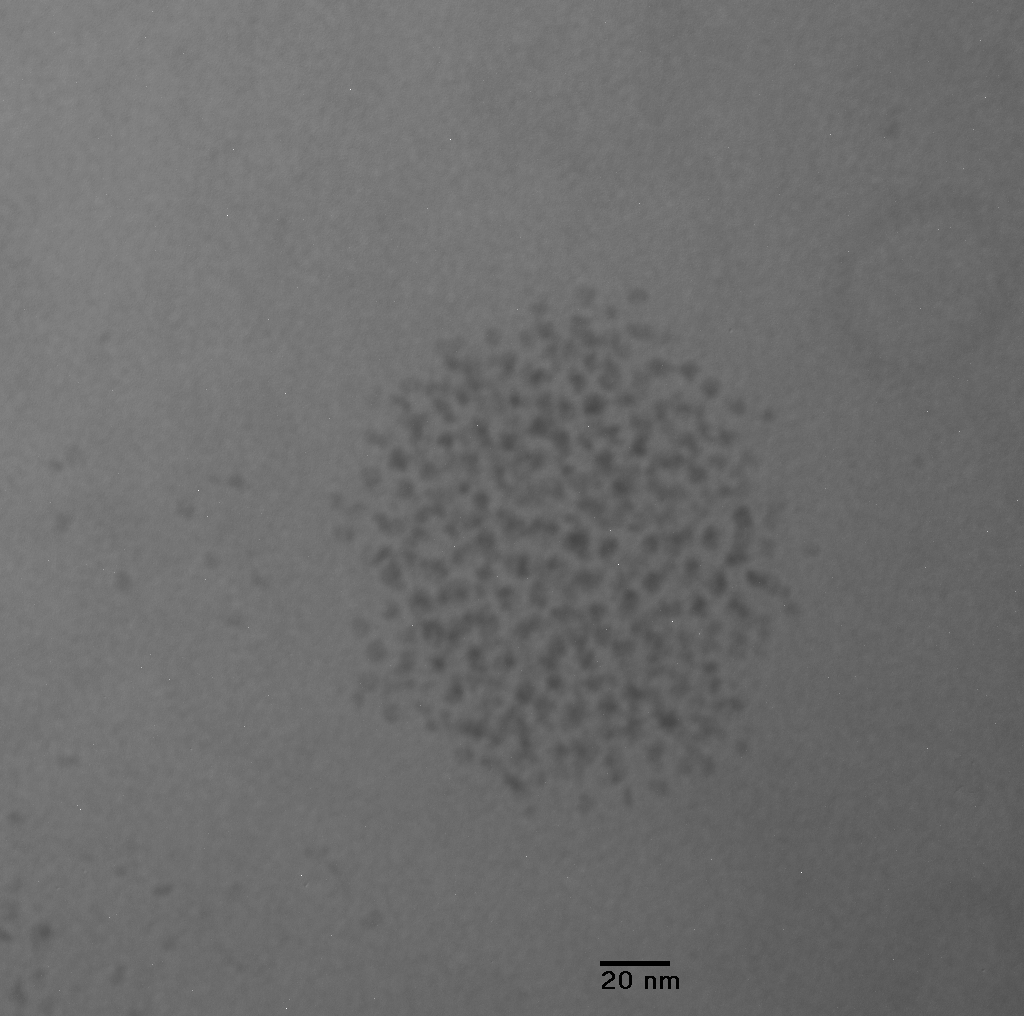

Supplement: Figure S7 — Electron micrograph of Qdot cluster imaged in midplane. Thin section taken from a lymph node. The lymph node was fixed and embedded in Epon without staining, then 20 nm sections were cut and imaged using an electron microscope. This aggregate is typical of those seen so far. Diameter of the cluster is approximately 120 nm. (TIF) [file pone.0051995.s007.tif]

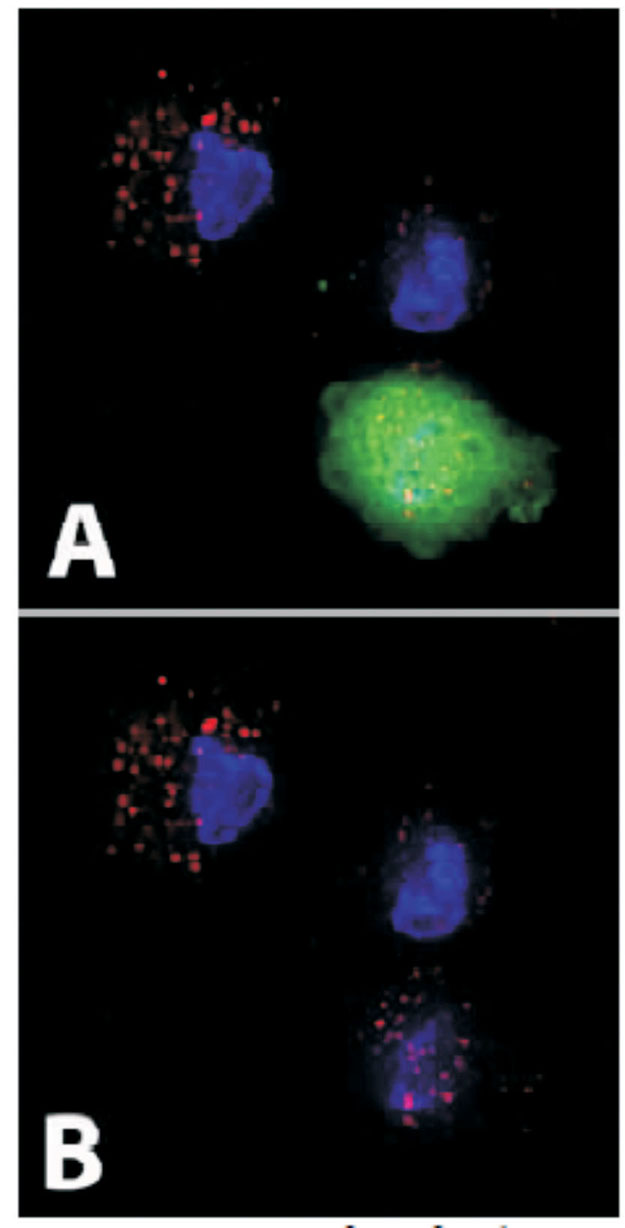

Supplement: Figure S8 — Transfer of Qdots between dendritic cells (DC’s). To test the possibility that Qdots can be passed among cells as they are transported through the tissue to eventually reach the draining lymph nodes, rather than just drifting between cells, we used monocyte-derived dendritic cells (MDDCs) as a model for the passing of Qdots between cells. One aliquot of activated MDDC was stained with carboxyfluorescein diacetate, succinimidyl ester (CFSE, Invitrogen, Molecular Probes, Eugene, OR), washed, and then mixed with Qdots for 15 minutes, followed by washing with PBS. The Qdot-loaded DCs were then mixed with unlabelled DCs for 15 minutes before plating onto coverslips. The results in Figure S8 show that the Qdots were transferred from one labeled population to the other during co-culture. Note that in Panel B, CFSE emission is not shown in order to reveal the remaining Qdots in the CFSE-stained cell. Thus, one way that the Qdots move through the submucosa may be passage between cells within the tissue. Green, CFSE-labeled DC’s; red, Qdots; Blue, DAPI. Transfer of the Qdots to the unlabeled DC’s is readily apparent. Panel A shows three colors while panel B (no CFSE displayed) shows that the CFSE labeled cell DC shown in A still retains abundant Qdots. (TIF) [file pone.0051995.s008.tif]
